# Recent advances in delivery of veterinary DNA vaccines against avian pathogens

**DOI:** 10.1186/s13567-019-0698-z

**Published:** 2019-10-10

**Authors:** Seyed Davoud Jazayeri, Chit Laa Poh

**Affiliations:** Centre for Virus and Vaccine Research, School of Science and Technology, Sunway University, 47500 Subang Jaya, Selangor Malaysia

## Abstract

Veterinary vaccines need to have desired characteristics, such as being effective, inexpensive, easy to administer, suitable for mass vaccination and stable under field conditions. DNA vaccines have been proposed as potential solutions for poultry diseases since they are subunit vaccines with no risk of infection or reversion to virulence. DNA vaccines can be utilized for simultaneous immunizations against multiple pathogens and are relatively easy to design and inexpensive to manufacture and store. Administration of DNA vaccines has been shown to stimulate immune responses and provide protection from challenges in different animal models. Although DNA vaccines offer advantages, setbacks including the inability to induce strong immunity, and the fact that they are not currently applicable for mass vaccination impede the use of DNA vaccines in the poultry industry. The use of either biological or physical carriers has been proposed as a solution to overcome the current delivery limitations of DNA vaccines for veterinary applications. This review presents an overview of the recent development of carriers for delivery of veterinary DNA vaccines against avian pathogens.

## Introduction

Vaccines have been effective against infectious diseases in animals and have successfully controlled and/or eradicated major animal pathogens. Based on the guidelines proposed by the Royal Society’s report on infectious diseases of livestock in 2002, UK, the characteristics of an ideal vaccine are:

- provides broad-spectrum protection against all isolates of the virus in all the affected species, preventing virus carriage and the possibility of shedding and transmission;
- stimulates the level of immunity necessary to drive effective and long-lasting immune responses;
- inexpensive to manufacture and simple to administer;
- in the case of live attenuated vaccines, reversion to virulence has to be avoided;
- has a long shelf life and is heat stable;
- allows discrimination between infected and vaccinated animals; and
- provides strong levels of maternal immunity.

Nevertheless, there is no single vaccine that has all the above characteristics. The use of vaccines to control disease is based on assessing the risks and evaluating the benefits following vaccination. Generally, genetic vaccines are composed of either DNA (as plasmids) or RNA (as mRNA) that is taken up and translated into proteins by cells of the vaccinated animals. Since there are limited reports on RNA vaccines compared to the extensive literature on DNA vaccines, genetic vaccines are generally referred to as plasmid DNA antigen-expression systems. Genetic immunization, also termed DNA immunization, is a recent vaccine technology utilizing eukaryotic expression vectors encoding antigens [1].

Wolff et al. first demonstrated that direct intramuscular (IM) injection of plasmid DNA was able to generate the expression of the plasmid-encoded antigen in a murine model [2]. To date, DNA vaccines have been successfully licensed for use against West Nile virus in horses [3], infectious haematopoietic necrosis in schooled salmons [4], and canine melanoma in dogs [5], as well as Clynav against pancreas disease infection in Atlantic salmon [6]. Moreover, the first commercial DNA vaccine against H5N1 in chickens has recently been conditionally approved by the United States Department of Agriculture (USDA), which targets highly pathogenic H5 avian influenza [7].

The first DNA vaccine that was studied in poultry in 1993 was directed against avian influenza virus (AIV) [8]. Immunization with DNA vaccines has had some success that could be attributed to their advantages over conventional vaccines. Despite the success of some DNA vaccines in small animal models in veterinary applications, there are still limitations in plasmid delivery and lack of immunogenicity in large animal models. To improve the immunogenicity of DNA vaccines, adjuvants have been co-administered in vivo with DNA vaccines. It is also possible to incorporate an immunomodulatory adjuvant into the plasmid and co-express the adjuvant gene. Immunomodulatory genes, including cytokines (IL15, IL18) [9], Esat-1 [10], MDP-1 [11], HMGB1ΔC [12] or HSP70 [13, 14], were found to enhance the humoral and cell-mediated immunity of AIV DNA vaccines. In addition, recent advances in the optimization of antigens carried in plasmids [15]; novel delivery methods, such as electroporation [16] or jet injections [17]; targeting of antigens to antigen-presenting cells (APCs) [18]; and co-delivery with biological [19] and nanoparticle [20] carriers have led to a substantial improvement in DNA vaccine efficacy in poultry.

**Figure 1:**
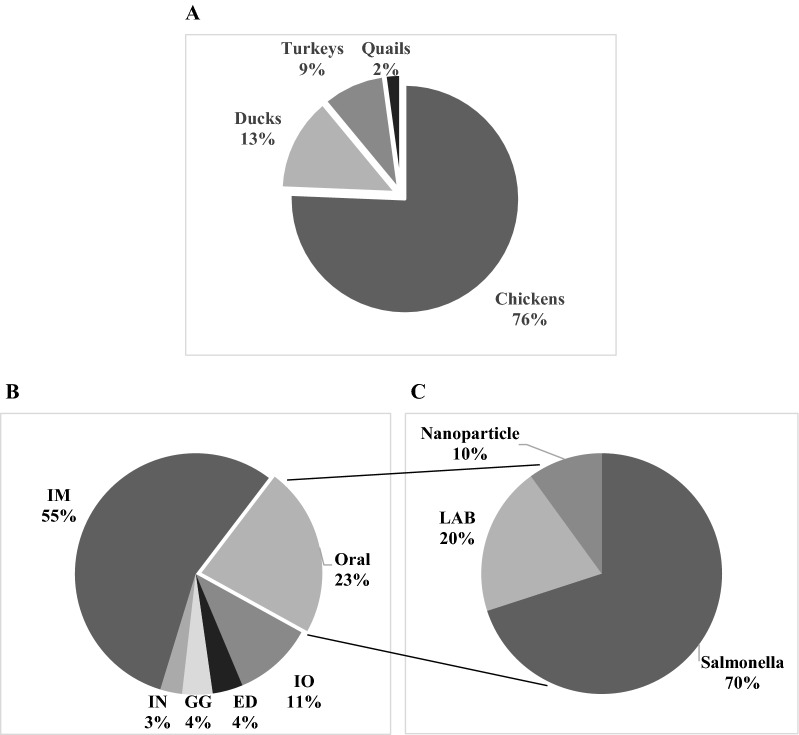
**Different poultry DNA vaccination models, routes and carriers. A** Relative proportions of DNA vaccine studies performed in poultry. **B** Relative proportions of different routes of administration in poultry. C *Salmonella* spp., LAB spp. and nanoparticles play major roles in oral delivery of DNA vaccines. IM: intramuscular, IO: in ovo, ED: eye drop, IN: intranasal, GG: gene gun, LAB: lactic acid bacteria.

Poultry DNA vaccines have been developed against several viral, bacterial and protozoan diseases. Promising results have been obtained and full protection (100%) elicited against poultry diseases, such as AIV in chickens and quails, duck Tembusu virus (DTMUV), infectious bursal disease virus (IBDV) and Newcastle disease virus (NDV) in chickens (Table 1). Based on the data summarized in Table 1, approximately 76% of poultry DNA vaccine studies were trialed in chickens, 13% in ducks, 9% in turkeys and just 2% in quails (Figure 1A). The efficacy of poultry DNA vaccines is affected by the age of the hosts, number of vaccination(s), vehicles and adjuvants, different routes of delivery and immunity against different pathogens (Table 1). Low in vivo efficacy contributed by factors such as the delivery system has always been the challenge for developing DNA vaccine utilization in poultry. Thus, this review is aimed at discussing the development of delivery systems for DNA vaccines in poultry. The benefits and pitfalls of using each delivery system will be discussed.

**Table 1:** **Efficacy of DNA vaccines against poultry pathogens delivered by different carriers**

Pathogens	Host/day of vaccination	Vehicle	Target antigen/adjuvant	Route of vaccination	Immune responses	Protection	Ref.
AIV	Chicken/3 and 6 week	Naked plasmid	H6 + Kozak sequence	IM	Anti-HA Ab	Not tested	[32]
AIV	Chicken/10, 24 and 38 day	Naked plasmid	H5 and HSP-70	IM	Anti-HA Ab	Not tested	[13]
AIV	Chicken/7, 21 and 35 day	Naked plasmid	H5 and Esat-6	IM	Anti-HA Ab	Not tested	[10]
AIV	Chicken/1 and 14 day	Naked plasmid	N1 and IL15	IM	Anti-N1 Ab/CD4 and CD8	Not tested	[9]
ILTV	Chicken/3 and 5 week	Naked plasmid	gB and IL18	IM	Anti-ILTV Ab and CD4 and CD8	80%	[84]
IBV	Chicken/7, 21 and 35 day	Naked plasmid	S1, N, and M	IM	IBV-neutralizing Ab and CD4/CD8	90%	[85]
NDV	Chicken/21, 36 and 46 day	Naked plasmid	HN, F, and IL4	IM	Anti-NDV Ab and cellular immune responses	40%	[34]
AIV	Chicken/3, 7 and 11 week	Naked plasmid + liposome	H5	IM	Anti-HA Ab	Not tested	[27]
AIV	Chicken/3 and 6 week	pHEMA	H6	IM	Ab response	Reduced virus shedding	[86]
*C. psittaci*	Turkey/1 and 3 week	Branched PEI	OmpA	IM	IgG and increased CD4/CD8 rate response	Reduced *C. psittaci* shedding and shortened the period of clinical signs	[77]
Anatid herpesvirus 1	Duck/4 week	Chitosan and liposome	gC	IM	Rapid and extensive plasmid distribution in duck tissues	Not tested	[87]
NDV	Chicken/1 week	Lipofectin	F and HN	IM	Anti-F Ab	80%	[88]
Coccidiosis	Chicken/1 and 2 week	Naked plasmid	Gam56 from *Eimeria maxima*	IM	Ab and lymphocyte proliferation	No chicken died from *E. tenella* challenge in any group	[89]
IBDV	Chicken/2 and 4 week	Naked plasmid	VP2 + HSP70	IM	Anti-VP2 Ab and lymphocyte proliferation	100%	[14]
CIA	Chicken/3, 5 and 7 week	Naked plasmid	VP1 and VP2 + HMGB1ΔC	IM	Anti-CIA Ab and CD8	Not tested	[12]
NDV	Chicken/30 and 44 day	Chitosan nanoparticles	F	IM/IN	IgA/IgG and lymphocyte proliferation	IM: 80% IN: 100%	[90]
REV	Chicken/3 and 6 week	Naked plasmid	gp90 + WPRE	IM	Lymphocyte proliferation, Ab response and IL-4 and IFN-γ	87%	[91]
*P. multocida*	Chicken/4, 6 and 8 week	Naked plasmid	OmpH and OmpA	IM	Induction of CD8 T cells, high serum Ab titres, and IFN-γ	70%	[92]
Coccidiosis	Chicken/2 and 3 week	Naked plasmid	TA4 and chicken IL-2	IM	Humoral response	~80%	[93]
Anatid herpesvirus 1	Duck/4 week	Naked plasmid + chitosan DNA and liposome	gC	IM	Extensive plasmid distribution in duck tissues	Not tested	[87]
DEV	Duck/10 and 12 week	Naked plasmid	gD and B	IM	Stimulated a high frequency of CD4, CD8 T cells and neutralizing antibody	70%	[94]
TCoV	Turkey/1, 7 and 21 day	Naked plasmid +PEI and sodium hyaluronate	4F/4R	IM	Anti-TCoV S Ab and VN titre	Decrease in clinical signs from 5/5 to 1/5 or 2/5	[95]
Coccidiosis	Chicken/4, 14 and 21 day	Naked plasmid	EtMIC2 + IL-18	IM	Anti-EtMic2 Ab, CD8/CD4 T cells	No chicken died from *E. tenella* challenge in any group	[96]
AIV	Quail/3, 6 and 9 week	Naked plasmid	H5	IM	Anti-HA Ab	100%	[21]
NDV	Chicken/30 and 44 day	Nano-chitosan	F	IM/IN	IgG and IgA and lymphocyte proliferation	80% (IM); 100% (IN)	[26]
IBDV	Egg embryonation on day 18 and at 1 week	Naked plasmid/killed vaccine	VP2, VP3, VP4 + killed virus booster	IO/IM	Anti-IBDV Ab and lymphocyte proliferation	100%	[43]
DPV	Duck/3 week	Naked plasmid +gold particles	gC	IM + GG	Increase the numbers of CD4, CD8 T cells and neutralizing antibody	Not tested	[97]
AVA	Chicken/6 and 13 day	*S. typhimurium* SL7207	pVAX-rC	Oral	Ab response	66.7%	[66]
AIV	Chicken/2 and 4 week	*S. typhimurium* SL7207	H1	Oral	Anti-HA intestinal mucosal IgA response and lymphoproliferation	100% (combined with killed vaccine)	[65]
Coccidiosis	Chicken/3 and 17 day	*S. typhimurium* SV4089	5401	Oral	Ab and cellular immune responses	55–57.5%	[62]
IBDV	Chicken/7 and 12 day	*S. typhimurium* SV4089	VP2/4/3	Oral	Ab response	73.3%	[61]
AIV	Chicken/1 day	*S. typhimurium* SV4089	H1	Oral	Anti-HA Ab and CD4/CD8 T cells	Not tested	[19]
CAV	Chicken/2, 4 and 6 week	*L. acidophilus*	VP1	Oral	Anti-CAV Ab/cell mediated responses	Not tested	[71]
IBDV	Chicken/7 and 14 day	Transgenic *E. coli* DH5α	VP2	Oral	Anti-IBDV Ab	95.4%	[54]
AIV	Chicken/1 day	NanoAg-poly(ethylene glycol)	H5	Oral	Anti-HA Ab and CD4/CD8 T cell response	Not tested	[20]
AIV	Chicken/10 and 24 day	Lactobacillus (LDL17-pH)	H1	Oral	Anti-HA mucosal IgA and serum IgG l, IFNγ, TLR-2, and AvBD-9 in the PPs and CTs	60%	[72]
DEV	Duck/3 times per day for 7 day	*S. typhimurium*	UL24 + labile enterotoxin B subunit	Oral	Serum IgA and IgG, DEV-specific mucosal IgA and anti-NA Ab	60-80%	[67]
DTMUV	Duck/8 and 24 day	*S. typhimurium* SL7207	prM and E	Oral	Specific antibody against the E protein and VN	100%	[68]
IBDV	Chicken/2 and 16 day	*S. enterica* serovar Typhimurium	S1 and N	Oral/IN	Humoral and mucosal immune responses	70%	[25]
*C. jejuni*	Chicken/1, 15 and 29 day	Nano-chitosan	Protein FlaA	IN	IgG and intestinal mucosa IgA responses	Not tested	[79]
*M. gallisepticum*	Chicken/2 week	*M. gallisepticum* ts-11	ts-11 C3 and IFN-γ	ED	Cell-mediated immunity	Not tested	[98]
IBDV	Chicken/3, 5 and 7 week	PLGA	VP2	ED	Stimulation of CD4 and CD8 T cells, high level of IgG	80%	[78]
AIV	Chicken/1 and 28 day	Naked plasmid	H7	IV, IP, and SC	Anti-HA Ab	50%	[99]
AIV	Chicken/3 week	Naked plasmid	H5	GG	Anti-HA Ab	100%	[16]
AIV	Duck/5 and 8 week	Naked plasmid	H5	External thigh muscle, Medi Jector Vision	NV Ab	Not tested	[100]
NDV and IBDV	Egg embryonation at 14–18 day	Plasmid/neutral lipid/DMSO	HA, VP2, VP4 and VP3	IO	Expression of viral protein	Not tested	[36]
NDV	Egg embryonation at 18 day	Naked plasmid	F and HN	IO	Anti-HA Ab	28%	[42]
IBV	Egg embryonation at 18 day	Naked plasmid/IFNα	gS	IO	Humoral immune responses	89%	[101]
ILTV	Egg embryonation at 18 day	Naked plasmid	CpG	IO	IgM, KUL01, and CD8 and CD4 cells	~40%	[41]

Ref: references, S1: spike protein, N: nucleocapsid protein, M: membrane protein, Ab: antibody, ILTV: infectious laryngotracheitis virus, g: glycoprotein, pHEMA: poly(2-hydroxyethyl methacrylate), REV: reticuloendotheliosis virus, TCoV: turkey coronavirus, DPV: duck plague virus, AVA: avian viral arthritis, PPs: Peyer’s patches, CTs: caecal tonsils, VN: virus neutralization, GG: gene gun, IV: intravenous, IP: intraperitoneal, SC: subcutaneous, d: day, wk: week.

## Routes of administration for DNA vaccine delivery

Effective DNA vaccine delivery is required to induce a strong and long-lasting immune response that can produce high and sustained levels of antigen production at targeted sites. Delivery routes of DNA vaccines can be generally grouped into those that are mucosal or systemic. Relative proportions of different administration routes of inoculation in poultry were calculated from the data summarized in Table 1 and presented in Figure 1B. The most extensively used routes for the delivery of poultry DNA vaccines include IM (55%), oral (23%), in ovo (IO) (11%), eye drop (ED) (4%) and intranasal (IN) (3%) (Figure 1B). Although some new delivery methods and routes are under development or being tested in poultry, conventional IM injection is still considered the dominant DNA vaccine delivery route. The majority of poultry DNA vaccines (approximately 55%) were applied as naked DNA through IM injection into the leg, chest or thigh muscles of poultry, and some promising results have been obtained. Full protection against a highly virulent H5N1 AIV infection was elicited in quails by IM immunization of a DNA vaccine encoding the H5 gene [21]. Ideally, DNA vaccine delivery should not be invasive [22]. However, most of the parenteral routes commonly used were needle-based deliveries and thus might cause complications in vaccinated chickens [23]. Compared with the parenteral routes, oral administration in poultry is faster and much easier to administer for mass application without requiring highly trained manpower and no risk of needle-stick injury or cross-contamination [24]. Oral immunization is able to induce mucosal immune responses and was performed as the second most popular route, with approximately 23% of poultry vaccinations. IO, which is specific to poultry, is the third most popular route of vaccination, at approximately 11% (Figure 1B).

Encapsulation of naked DNA with a carrier has been proposed as a solution to improve the controlled release of antigens that could increase the efficacy of DNA vaccines. Regardless of live, attenuated, killed or DNA vaccines, noninvasive vaccinations, including IN and oral delivery, could reduce stress, pain and cost of vaccinations and increase the safety of vaccination in large flocks of birds.

Furthermore, successful IN and oral delivery tend to raise better mucosal immunity than the other routes against poultry respiratory viruses, such as infectious bronchitis virus (IBV) [25], NDV [26], and AIV [19]. Thus, the design of carriers should help improve the efficacy and stability of DNA vaccines for IN or oral delivery. The carrier must be able to resist degradation and attack by the immune system and have sufficient safety profiles to become a successful delivery system.

## Vectors for delivery

Regardless of the choice of route, the low efficiency of traditional naked DNA vaccines has always been considered one of the main obstacles. To overcome this problem, improved expression vectors with more efficient promoters and the use of adjuvants were proposed to improve the efficacy of DNA vaccines in poultry. Lee et al. demonstrated that the use of the pCI-neo HA plasmid with the cytomegalovirus (CMV) promoter could effectively boost the antibody response against influenza virus in chickens [27]. Most of the plasmids that have been successfully used for poultry DNA vaccine development were mainly the same as those used in mammalian DNA vaccines, and few plasmids, such as pCAGGS (antigen transcription is under control of the chicken β-actin promoter), were specifically developed and used for poultry applications [28].

Codon optimization is based on the selection of codon triplets that have the highest tRNA utilization frequency in the cytoplasm, which can increase translation rates and mRNA stability. Successful DNA vaccination requires high expression of the antigenic gene(s) in the host, and this method is usually used to elicit foreign protein production [29]. The Kozak sequence plays a significant role in the initiation of a translation process in mammalian cells by increasing the chance of ribosome recognition of the AUG start codon in the transcription process [30].

In addition, the Kozak sequence was also found to enhance the expression of a DNA vaccine after immunization. However, the efficacy of a DNA vaccine with the Kozak sequence for both Marek’s disease (MD) and AIV was not well supported in two reports [31, 32]. Oligodeoxynucleotides rich in cytosine-guanosine deoxynucleotide (CpG) motifs were also found to enhance the innate immunity of chickens and effectively protected (~80%) against *S. typhimurium* septicaemia upon challenge [33]. In addition to using improved expression vectors and promoters, the development of multivalent DNA vaccines enhanced cell-mediated immunity. Sawant et al. constructed a bivalent DNA vaccine simultaneously expressing the HN and F antigens of NDV with the chicken immunomodulatory IL-4 gene. Chickens inoculated via the IM route displayed an increase in NDV-specific antibodies and cell-meditated immunity. The DNA vaccine conferred protection to 40% of chickens against NDV upon challenge [34].

Fusion of the *M. tuberculosis* HSP70 or Esat-6 genes with the H5 gene of AIV H5N1 was also found to enhance the antibody response in chickens [10, 13]. In addition to the fusion of two genes, Lim et al. demonstrated that co-delivery of N1 and IL-15 in 2 different plasmids induced higher humoral and cell-mediated immunity in chickens than vaccination with N1 alone [9]. Coexpression of chicken IL-2 and IL-7 enhanced the humoral and cell-mediated immunity as well as the protective efficacy of a VP2-expressing DNA vaccine against IBDV in chickens [35]. In addition, the DNA adjuvant neutral lipid with DMSO was reported to be suitable for IO vaccination with NDV and IBDV viral proteins [36]. Progress has been made towards the development of many monovalent DNA vaccines in poultry medicine, although the desirable practical farm DNA vaccine should be effective against multiple species. Novel multivalent T cell epitope DNA vaccines against four Eimeria species were constructed, and animal experimentation showed effective protection against all four species, *E. tenella*, *E. necatrix*, *E. maxima* and *E. acervuline*, in chickens [37].

IO delivery of CpG DNA has been shown to reduce bacterial infections with *S. enteritidis*, *S. typhimurium* and *E. coli* in chickens [38, 39]. It was found to mediate an antiviral response against influenza, which correlated with a macrophage response in the lungs [40]. In another trial, IO delivery of CpG DNA increased recruitment of IgM, KUL01, and CD8 and CD4 T cells at day 1 post-hatching in the trachea, lungs, duodenum, large intestine, spleen and bursa of chickens [41]. However, these modifications could only partially solve the low efficacy of DNA vaccines because the APCs were still not specifically targeted, and the encoded antigens were not delivered to the target site to produce sufficient mucosal or organ-specific immunity.

The efficacy of protection conferred by naked plasmids, with a few exceptions, was lower than 100%. For example, a naked plasmid carrying the F and HN genes of NDV, when administered through the IO route, could only confer protection to 28% of chickens [42]. The efficacy conferred by other naked plasmids carrying antigens against AIV, ILTV, IBV, REV, and DEV was reported to range from 50 to 90% protection. However, in another example, a naked plasmid carrying the H5 antigen against AIV was able to confer 100% protection when delivered by the gene gun (GG) in chickens (Table 1). Park et al. demonstrated that priming with a DNA vaccine encoding the VP2, VP3, and VP4 antigens through the IO route and boosting with IM injection of a killed IBD vaccine completely protected chickens against a highly virulent IBDV [43].

## Biological carriers

Bacteria have been described as “tiny programmable robot factories” for use in the delivery of DNA vaccines against various diseases (viral, bacterial and parasitic) [44]. The first report of in vitro gene transfer from bacteria to mammalian cells was reported over 30 years ago by Walter Schaffner, where tandem copies of the SV40 virus genome were transferred into co-cultured mammalian cells using laboratory strains of *E. coli* [45]. Bacteria-based DNA delivery systems are able to replicate in the host and, by carrying their own immunostimulatory factors, could elicit immune responses not only against the plasmid-encoded foreign antigens but also against the bacterial carrier itself [46, 47]. Briefly, bacteria as poultry DNA vaccine carriers are divided into gram-positive (non-pathogenic) and gram-negative strains (attenuated pathogenic bacteria).

### Gram-negative bacteria as potential carriers for DNA vaccines

**Figure 2:**
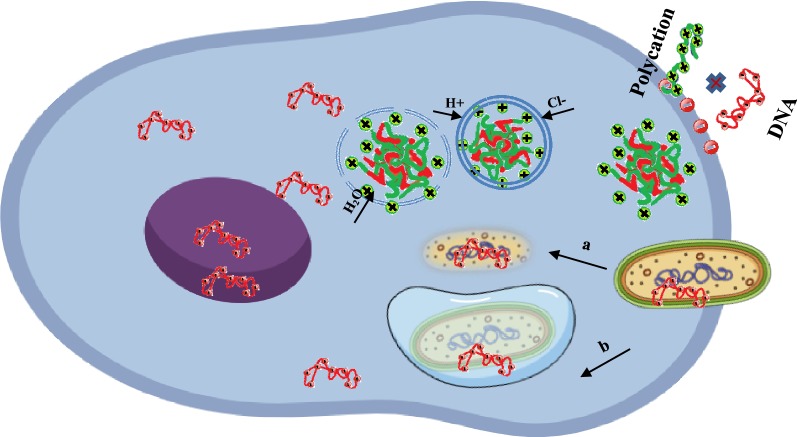
**Principles of different carriers for DNA vaccine delivery to host cells.** (1) Bacterial-mediated delivery. (a) Bacteria, such as *L. monocytogenes*, *E. coli,* and *S. flexneri*, carrying a recombinant plasmid invade host cells, escape from the vacuole system, then die in the cytosol and release the plasmid [102]. (b) Bacteria, such as Salmonella, first invade the host cells, remain in the vacuole, then die due to metabolic attenuation and release the recombinant plasmid into the cytosol [102]. (2) Polycations are able to compress the molecular size of plasmids into compact structures by converting the negative charges to positive. The high surface cationic charge of an encapsulated plasmid mediates both size condensation and buffering capacity that diminish the requirement for the addition of endosomolytic agents. Buffering leads to osmotic swelling, membrane lysis and subsequent plasmid release [103].

There are some gram-negative pathogenic bacteria, such as *E. coli* and *Salmonellae* species, that have been isolated and used for DNA vaccine delivery in poultry. The trafficking of intracellular gram-negative bacteria can be divided into intraphagosomal and intracytosolic pathways. With regard to localized infections of bacteria in host cells, enteropathogenic bacteria are used as DNA vaccine carriers, and these can be divided into (1) extracellular pathogens, such as *E. coli* or *Yersinia* spp. (*Y. pseudotuberculosis* and *Y. enterocolitica*); (2) intraphagosomal bacteria, such as *Salmonella* spp.; and (3) intracytosolic bacteria, such as *Shigella* spp. and *Listeria monocytogenes* (Figure 2) [48–53]. Oral immunization of chickens with a DNA vaccine encoding the VP2 gene of IBDV carried by a transgenic *E. coli* DH5α resulted in 95.4% protection [54].

#### Salmonella

*Salmonella*, as a non-host-specific intracellular bacterium, is commensal in poultry and can persist in the gastrointestinal (GI) tract. Different *Salmonella* serotypes derived from a wide range of hosts can infect poultry [55]. Therefore, avian species can be infected by host-specific and non-host-specific *Salmonella* serotypes. Avian systemic salmonellosis is characterized by three separate phases: invasion of *Salmonella* via the GI tract, establishment of infection in macrophages and subsequent clearance of the infection by the immune system. Otherwise, the birds develop the subclinical phase of salmonellosis and die [56].

*Salmonella* has a close relationship with poultry and has been evaluated as a live carrier for inducing protective responses to a wide variety of infections due to its ability to improve the efficacy of a vaccine through induction of mucosal and internal organ immunity [57]. *Salmonella* can invade, survive and multiply in APCs (macrophages), which are the critical characteristics of *Salmonella* as a carrier for vaccine development. Moreover, live attenuated *Salmonella* can release transformed plasmids through an unknown mechanism into the eukaryotic cytoplasm (Figure 2) [58, 59]. Hence, *Salmonella* is a promising carrier in poultry DNA vaccine development.

*Salmonella* is able to orally infect animals and humans. Following ingestion, a proportion of the bacteria can resist the low pH of the GI tract and reach the ileum and the caecum; then, *Salmonella* can invade the mucosa by multiplication in the sub-mucosa and in Peyer’s patches (PPs). In young birds with an immature immune system, extensive replication of bacteria occurs in the caecum [60].

Various attenuation methods have been applied to reduce the pathogenicity of *S. typhimurium*, which retained their invasive ability and could deliver a heterologous plasmid into mammalian cells. *S. typhimurium* SV4089, a double mutant (Dam^−^ and PhoP^−^) of wild-type *S. typhimurium* SL1344, is an attenuated *Salmonella* strain that has been used extensively as a carrier for DNA vaccines in different animal models. Studies have shown that *S. typhimurium* SV4089 is not orally pathogenic to chickens at a dose level as high as 10^10^ cfu/mL [61, 62], while the oral LD50 of wild-type SL1344 in chickens is ~10^4^ cfu/mL [63]. The live attenuated *S. typhimurium* SV4089 provided a unique alternative in terms of safety and in vitro and in vivo stability of transfected plasmids. DNA vaccines are inexpensive to produce in large doses and are easily detected and monitored after oral inoculations into the host. Furthermore, attenuated *S. typhimurium* SV4089 was able to invade and pass through the various organs of inoculated chickens, such as the liver, spleen, and caecum, without showing evidence of systemic infection [64].

Attenuated *S. typhimurium* has been used as a carrier for DNA vaccines against different pathogens in poultry. In one study, oral administration of chickens with the attenuated *S. typhimurium* SV4089 containing pcDNA3/*E. tenella* 5401 antigen showed strong humoral and cellular immunity, with partial protection (55–57.5%) against challenge from *E. tenella* [62]. Li et al. demonstrated that oral administration of chickens with the attenuated *S. typhimurium* SV4089 containing the complete polyprotein (VP2/4/3) of IBDV also offered 73.3% protection against challenge with a virulent IBDV [61]. In another study, Jazayeri et al. showed that a single oral immunization of chickens with 10^9^ cfu/mL *S. typhimurium* SV4089 containing a eukaryote expression vector encoding the haemagglutinin (HA) gene of H5N1 did not produce any clinical manifestations. Orally vaccinated chickens showed anti-H5 antibody production, increased CD4/CD8 T cell levels and mixed proinflammatory/Th1-like cytokine responses against AIV, which was important for viral clearance [19].

In addition to the attenuated *S. typhimurium* strain SV4089, another attenuated *S. typhimurium* strain, SL7207, was also studied as a carrier for DNA vaccines. Pan et al. showed that oral vaccination of white leghorn chickens with an HA DNA vaccine carried by the attenuated *S. typhimurium* SL7207 and boosted with a killed H9N2 vaccine was able to confer 100% protection against H5N1 following challenge, with no virus shedding or clinical signs [65]. Wan et al. used 1.0 × 10^10^ cfu/mL *S. typhimurium* SL7207 as a carrier for oral vaccination of chickens against avian reovirus (ARV) by using the σC protein. The results showed high levels of antibody production as well as protection of 66.7% of chickens against ARV challenge [66]. In addition to vaccinating chickens, oral DNA vaccination against duck enteritis virus (DEV) administered by the attenuated *S. typhimurium* SL7207 carrier co-expressing UL24 (core herpesvirus gene) and *E. coli* heat labile enterotoxin B subunit (LTB) as a mucosal adjuvant was able to induce effective systemic and mucosal immune responses and showed 60–80% protection of the ducklings [67]. Moreover, oral delivery of the *Salmonella* SL7207 strain carrying a DNA vaccine (pVAX1-SME) encoding the envelope proteins prM and E of DTMUV displayed strong immunogenicity and provided protection to 100% of ducks against DTMUV infection. Ducks orally vaccinated with this DNA vaccine were protected from lethal DTMUV infection. Oral administration of the DTMUV vaccine provided a fast vaccine delivery strategy and was economical for large-scale clinical applications [68]. Jiao et al. reported that a DNA vaccine encoding the S1 and N genes delivered by *S. enterica* serovar Typhimurium via the oral and IN routes could induce humoral and mucosal immune responses and conferred 70% protection against IBV in chickens [25].

### Gram-positive LAB

LAB (*Lactococcus*, *Streptococcus,* and *Lactobacillus*) are nonsporulating, have low G + C content and are non-pathogenic food-grade bacteria. They are an excellent candidate for functioning as adjuvants, immunostimulators and live antigen carriers to deliver antigens and cytokines at the mucosal level [69]. Dieye et al. characterized *L. lactis* as a potential vehicle for protein delivery (VP2 and VP3), serving as a live mucosal vaccine against IBDV in chickens [70].

Moreover, Moeini et al. showed that *L. acidophilus* carrying the VP1 protein of chicken anaemia virus (CAV)-induced neutralizing antibodies and Th1 cytokines against CAV in orally vaccinated chickens and suggested that *Lactobacilli* could also be used as a potential carrier for oral immunization of chickens [71]. In another study, Wang et al. demonstrated oral vaccinations of chickens with a recombinant lactobacillus (LDL17-pH), which expressed avian HA1 protein and could significantly increase the specific mucosal anti-HA1 IgA levels and anti-HA1 serum IgG levels. The chickens were protected at a level of 60% against lethal challenge with a H5N1 virus [72].

## Physical carriers

Physical approaches are the most commonly employed for DNA vaccine delivery. However, physical carriers need to successfully permeate the cell membrane of the target cell and release the DNA vaccine into the cytoplasm. The polycation-based delivery system is a promising approach for non-viral delivery because its molecular entity can be modified to fine tune and change its physicochemical properties. Since DNA is a large molecule (up to 1 μm in length), is negatively charged and, as a general rule, the plasma membrane of living cells is proportionately lipophilic and is also negatively charged, it is expected that the cell membrane could act as a barrier for large-sized polynucleotides. In addition, naked DNA associates poorly with the cell membrane [73, 74].

Polycations have been used to address the problems of changing the negative charges of nucleotides to positive and compressing the molecular size of the plasmid into compact structures that are necessary for transfecting nucleotides into most types of eukaryotic cells (Figure 2). It is likely that an encapsulated DNA with a slightly positive charge could interact electrostatically with the cell membrane and then be internalized. The adsorption of DNA to the surface of positive polymers during electrostatic interactions plays a major role in improving the efficiency of DNA vaccines. More significantly, cationic polymers on the nano-scale have received heightened attention because they further enhanced the chemical stability of DNA vaccines and induced enhanced immune responses since the uptake of nanoparticle carriers with DNA vaccines into immune cells, such as dendritic cells (DC), was highly effective [75].

One of the cationic nano-polymers is nano-polyethyleneimine (PEI), which is able to electrostatically bind plasmid DNA (pDNA) and condense it into positively charged molecules, which can be taken up by cells more effectively than naked DNA. Among the PEI types, branched PEI was found to be more effective and stable than linear PEI in delivering the *omp*A gene to protect against *Chlamydophila psittaci* infection. Branched PEI was able to activate both humoral and cell-mediated immunity post vaccination. This effect might be contributed by PEI to deliver the DNA that activated APCs, such as DCs [76]. However, the vaccination could only help to reduce *C. psittaci* shedding and shorten the period of clinical signs in infected chickens but failed to raise sufficient protection against challenge [76, 77]. In addition to PEI, poly(lactide-co-glycolide) (PLGA) was also found to be effective, as it could prolong and promote sustainable release of DNA, which was taken up by APCs. Both PEI and PLGA were found to be effective in delivering the VP2 gene of IBDV and elicited both humoral (IgG) and cell-mediated immunity (CD4/CD8). Negash et al. used PLGA-PEI macroparticles adsorbed with a recombinant plasmid carrying the VP2 gene and showed that immunization of chickens could improve the efficacy of the IBDV DNA vaccine to prevent both morbidity and mortality in up to 80% of birds [78].

Other biomaterials, such as chitosan, have also been used as carriers for poultry DNA vaccines. White leghorn chickens were used as a model for IN immunization with chitosan/DNA nanoparticles, which carried the FlaA gene of *C. jejuni*. They produced significantly increased levels of IgG antibodies against *C. jejuni* and intestinal mucosal antibodies (IgA) [79]. Meanwhile, Zhang et al. successfully prepared spherically shaped chitosan nanoparticles with mean diameters between 100 and 200 nm and a positive surface charge, which could protect DNA against DNase I degradation. Plasmid DNA containing the HN and chicken IL-2 genes encapsulated with chitosan nanoparticles showed improved DNA vaccine efficacy and elicited haemagglutination inhibition (HI) antibody titres and IFN-γ against NDV challenge in chickens [80]. Recently, Gong et al. also successfully developed chitosan nanoparticles (spherical shape and approximately 200 nm) to encapsulate a *ptfA*-DNA vaccine against *Pasteurella multocida*, with an encapsulation efficiency of 95.3%, and the formulation effectively resisted DNase degradation. IM vaccination of the encapsulated *ptfA*-DNA vaccine into 4-week-old chickens induced higher antibody concentrations and lymphocyte proliferation than naked DNA and conferred 68% protection, compared to the 56% achieved by naked DNA [81]. IN and IM immunizations of a DNA vaccine encoding the F antigen of NDV encapsulated with chitosan nanoparticles induced 100% and 80% protection in chickens, respectively [26].

In addition to higher solubility and penetration into the cell, nanoparticles also provided the flexibility to be conjugated with other nanomaterials to further increase the specificity and efficacy of delivery. Moreover, Jazayeri et al. prepared green silver nanoparticles (nanoAg) with poly-ethylene glycol for delivery of the H5 gene of AIV into primary duodenal chick cells. The results demonstrated that the nanoAg were able to completely encapsulate the DNA, protected the H5 gene against DNase I and transferred the complex into primary cells as early as 1 h after transfection [82]. Moreover, single oral administration of DNA/H5 plasmid encapsulated in nanoAg in chickens induced antibodies and cell-mediated immune responses as well as enhanced cytokine production [20].

Biodegradability, increased immunogenicity, flexibility in conjugation with other molecules, including antibodies to specify the target delivery, and no involvement of live organisms (viruses or bacteria) in physical carriers have supported their potential to overtake biological carriers in the delivery of DNA vaccines. However, their cytotoxicity, safety (induction of non-specific inflammation/allergic reaction) and capacity of DNA loading need to be further evaluated not only in vitro but also in field trials for veterinary vaccine delivery studies [83]. Currently, the most commonly used oral DNA vaccine delivery vehicles in poultry vaccination involve Salmonella species (70%), LAB (20%) and nanoparticles (10%) (Figure 1C).

## Conclusions

DNA vaccination provided a new and valuable approach to the development of poultry vaccines and offered advantages in flexibility of design, speed, simplicity of production, and the ability to elicit both cellular and humoral immune responses. DNA vaccines against influenza in poultry have been in development since 1993, and recently, the USDA conditionally approved the first DNA vaccine against H5N1 for chickens. DNA vaccines are amendable for stockpiling to control future influenza H5N1 outbreaks. The pandemic AIV strains have undergone antigenic shift or drift, which allows them to avoid immunity elicited by the poultry influenza vaccines. Recent AIV vaccine development studies have indicated the need for additional systemic vaccine challenge studies against highly pathogenic AIV. Moreover, full protection has been demonstrated against poultry diseases, such as DTMUV, IBD, and ND. DNA vaccines also suffer from several pitfalls where in vivo efficacy and stability are still problems. Additionally, a single DNA vaccination in poultry is often insufficient to induce robust humoral and cell-mediated immunity as well as confer full protection. Therefore, booster immunization is often required. Both biological and physical carriers, with their appropriate antigens and adjuvants, offer the possibility to overcome the disadvantages of DNA vaccines. Although DNA vaccines carrying different antigens have been delivered by different types of carriers and adjuvants, very few have been evaluated by challenges with the pathogens in question. Thus, additional in vivo field trials should be carried out to identify the efficiency and safety of the currently available carriers, antigens, and adjuvants to combat infectious diseases of veterinary pathogens.

## Abbreviations

LAB: lactic acid bacteria
IM: intramuscular
USDA: The United States Department of Agriculture
AIV: avian influenza virus
APCs: antigen-presenting cells
DTMUV: duck Tembusu virus
IBDV: infectious bursal disease virus
NDV: Newcastle disease virus
IO: in ovo
ED: eye drop
IN: intranasal
CMV: cytomegalovirus
IBV: infectious bronchitis virus
CpG: cytosine-guanosine deoxynucleotide
GI: gastrointestinal
HA: haemagglutinin
ARV: avian reovirus
DEV: duck enteritis virus
CAV: chicken anaemia virus
DCs: dendritic cells
PEI: polyethyleneimine
pDNA: plasmid DNA
PLGA: poly(lactide-co-glycolide)
HI: haemagglutination inhibition
nanoAg: silver nanoparticles
pHEMA: poly(2-hydroxyethyl methacrylate)
GG: gene gun
Ref: references
ILTV: infectious laryngotracheitis virus
g: glycoprotein
REV: reticuloendotheliosis virus
TCoV: turkey coronavirus
DPV: duck plague virus
AVA: avian viral arthritis
PPs: Peyer’s patches
CTs: caecal tonsils
VN: virus neutralization
S1: spike protein
N: nucleocapsid protein
M: membrane protein
IV: intravenous
IP: intraperitoneal
SC: subcutaneous
Ab: antibody
d: day
wk: week
